# SARS-CoV-2 Spike-Specific CD4+ T Cell Response Is Conserved Against Variants of Concern, Including Omicron

**DOI:** 10.3389/fimmu.2022.801431

**Published:** 2022-01-26

**Authors:** Alessio Mazzoni, Anna Vanni, Michele Spinicci, Manuela Capone, Giulia Lamacchia, Lorenzo Salvati, Marco Coppi, Alberto Antonelli, Alberto Carnasciali, Parham Farahvachi, Nicla Giovacchini, Noemi Aiezza, Francesca Malentacchi, Lorenzo Zammarchi, Francesco Liotta, Gian Maria Rossolini, Alessandro Bartoloni, Lorenzo Cosmi, Laura Maggi, Francesco Annunziato

**Affiliations:** ^1^ Department of Experimental and Clinical Medicine, University of Florence, Florence, Italy; ^2^ Infectious and Tropical Disease Unit, Careggi University Hospital, Florence, Italy; ^3^ Microbiology and Virology Unit, Careggi University Hospital, Florence, Italy; ^4^ Immunology and Cell Therapy Unit, Careggi University Hospital, Florence, Italy; ^5^ Flow Cytometry Diagnostic Center and Immunotherapy, Careggi University Hospital, Florence, Italy

**Keywords:** SARS-CoV-2, CD4, T cells, variants of concern (VOCs), cellular immunity, vaccination, natural infection

## Abstract

Although accumulating data have investigated the effect of SARS-CoV-2 mutations on antibody neutralizing activity, less is known about T cell immunity. In this work, we found that the ancestral (Wuhan strain) Spike protein can efficaciously reactivate CD4+ T cell memory in subjects with previous Alpha variant infection. This finding has practical implications, as in many countries only one vaccine dose is currently administered to individuals with previous COVID-19, independently of which SARS-CoV-2 variant was responsible of the infection. We also found that only a minority of Spike-specific CD4+ T cells targets regions mutated in Alpha, Beta and Delta variants, both after natural infection and vaccination. Finally, we found that the vast majority of Spike-specific CD4+ T cell memory response induced by natural infection or mRNA vaccination is conserved also against Omicron variant. This is of importance, as this newly emerged strain is responsible for a sudden rise in COVID-19 cases worldwide due to its increased transmissibility and ability to evade antibody neutralization. Collectively, these observations suggest that most of the memory CD4+ T cell response is conserved against SARS-CoV-2 variants of concern, providing an efficacious line of defense that can protect from the development of severe forms of COVID-19.

## Introduction

Immunological memory is achieved by either natural infection or vaccination and can guarantee long lasting protection from moderate to severe COVID-19, in case of SARS-CoV-2 encounter. For this reason, mass vaccination strategies are currently underway to speed up the process of immunization against SARS-CoV-2. However, the exponential propagation of SARS-CoV-2 worldwide has led to the emergence of viral variants with increased transmission capability. Between the end of 2020 and the beginning of 2021, several SARS-CoV-2 variants arose, rapidly spreading worldwide and replacing the original Wuhan strain. The B.1.1.7 (Alpha) variant emerged in the UK in September 2020, showed an increased transmissibility and substituted rapidly the ancestral strain in Europe and the US (1, 2). The B.1.351 (Beta) variant instead was originally described in South Africa in May 2020 (1, 3). The WHO classified these strains as variants of concern (VOC) on December 18, 2020. The B.1.617.2 (Delta) variant which was originally detected in India, was classified as VOC on May 18, 2021, and became rapidly the leading strain worldwide (1). Nowadays, the majority of COVID-19 cases are due to the Omicron variant, which arose in South Africa and was declared by the WHO as VOC on November 26, 2021 (4). The emergence of viral variants with increased transmissibility has been associated to increased numbers of COVID-19 cases (5). Amino acid mutations in viral proteins may mine the capacity of SARS-CoV-2 specific antibodies and T cells to recognize mutated proteins, thus potentially overcoming the immunological memory-mediated protection. Particular attention is given to mutations in the Spike protein, since it is crucial in the virus infection process and it is targeted by currently approved vaccines. Accumulating evidences have shown a relatively small impact of Alpha variant on the antibody neutralization capacity, while Beta variant is associated to a significant loss of neutralizing activity. These observations were obtained both on sera from recovered individuals and from vaccinated subjects (6). Convalescent and vaccinated sera showed reduced neutralizing antibody titers also against Delta (7). Recent evidences are now demonstrating that the antibody neutralization capacity is significantly reduced against Omicron both in previously SARS-CoV-2 infected and vaccinated subjects (8), although a third, booster, vaccine administration seems to partially restore the neutralizing capability (9). Indeed, Omicron is heavily mutated in the receptor binding domain of the Spike protein, thus explaining its increased immune evasion potential (10). As far as T cell recognition is concerned, there are few information regarding the effect of viral mutations. At a clinical level, BNT162b2 and mRNA-1273 mRNA vaccines (from Pfizer/Biontech and Moderna, respectively) and the viral vector based ChAdOx1 (AstraZeneca) vaccine retain high efficacy against Alpha variant (11), while a significant loss has been demonstrated for ChAdOx1 against Beta variant (12). In this manuscript, we investigated the capability of circulating CD4+ T cells derived from subjects who recovered from SARS-CoV-2 infection or who were vaccinated with BNT162b2, mRNA-1273 or ChAdOx1, to respond to SARS-CoV-2 variants of concern including the recently emerged Omicron.

## Results

**Figure 1:**
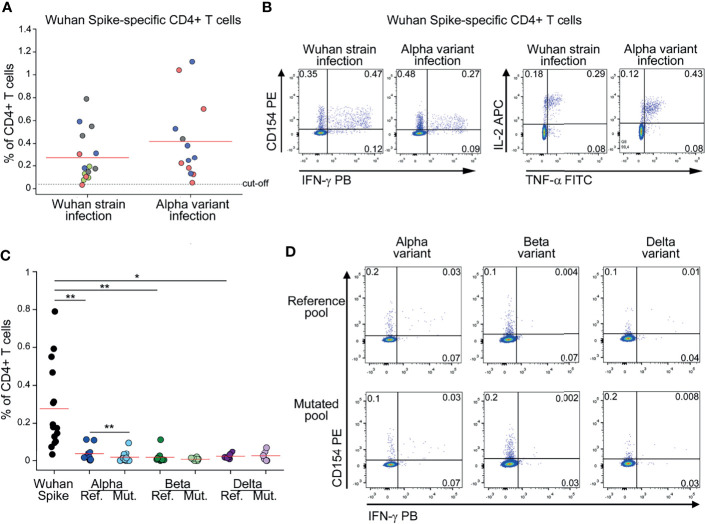
CD4+ T cell response to Spike in subjects with previous SARS-CoV-2 infection is minimally affected by mutations occurring in Alpha, Beta and Delta variants **(A)** Frequency of CD4+ T cells specific for Wuhan Spike protein in Wuhan strain- (15) or Alpha variant- (13) infected subjects. Mild COVID-19 patients are depicted in green, moderate in red, severe in blue, critical in gray. Representative FACS plots from one subject of each group are depicted in **(B)**. **(C)** Frequency of CD4+ T cells specific for Wuhan Spike, Alpha variant reference or mutated pool, Beta variant reference or mutated pool, Delta variant reference or mutated pool in subjects with previous Wuhan strain SARS-CoV-2 infection. Representative FACS plots showing CD4+ T cells reactive to Alpha, Beta and Delta reference and mutated pools from three distinct patients are depicted in **(D)**. Data in **(A, C)** are subtracted of background, unstimulated condition. Red lines in **(A, C)** represent mean values. Dashed line in **(A)** represents cut-off value. *p < 0.05; **p < 0.01, calculated with Wilcoxon-Signed Rank test.

To evaluate the frequency of circulating CD4+ T cells specific for Spike, we stimulated *in vitro* peripheral blood mononuclear cells (PBMC) with peptide pools spanning the entire ancestral (Wuhan strain) Spike sequence. Reactive CD4+ T cells were identified based on surface CD154 expression and production of at least one among IFN-γ, TNF-α and IL-2 cytokines (13, 14). Tested PBMC had been collected from subjects who had recovered from ancestral SARS-CoV-2 infection (before October 2020) or from subjects with laboratory confirmed SARS-CoV-2 Alpha variant infection. As shown in **Figures 1A, B**, we detected comparable frequencies of ancestral (Wuhan strain) Spike-reactive CD4+ T cells in subjects with history of ancestral- or Alpha-variant infection. This finding suggests that mutations occurring in Alpha variant do not affect the global CD4+ T cell capacity to recognize the ancestral Spike protein. To gain a better insight on the effect of SARS-CoV-2 mutations on CD4+ T cell response, we stimulated PBMC from subjects with previous ancestral SARS-CoV-2 infection also with peptide pools selectively spanning regions of the ancestral Spike protein that are mutated in the Alpha, Beta or Delta variant (Alpha, Beta or Delta reference pools). As shown in **Figure 1C**, frequencies of CD4+ T cells specific for Alpha, Beta or Delta reference pools were significantly lower than those specific for the entire ancestral Spike protein. This observation suggests that a minor fraction of the entire Spike-specific CD4+ T cell population is involved in the recognition of regions mutated in Alpha, Beta and Delta variants. Indeed, they represented 11%, 7% and 10% of total CD4+ T cell response to Spike, respectively. Then, we tested if the recognition of Alpha, Beta or Delta reference pools by CD4+ T cells is affected by mutations occurring in the respective variants. To test this, PBMC from subjects with previous ancestral SARS-CoV-2 infection were stimulated with Alpha, Beta or Delta reference pools and with the corresponding mutated pools. As shown in **Figures 1C, D**, mutations occurring in Alpha variant mildly affected CD4+ T cell response. On the contrary, mutations occurring in Beta and Delta variants did not significantly reduce CD4+ T cell recognition (**Figure 1C**).

Collectively, these data show that the majority of Spike-specific CD4+ T cell response is directed to non-mutated regions and, in any case, it is similar in Wuhan strain and Alpha variant infection.

**Figure 2:**
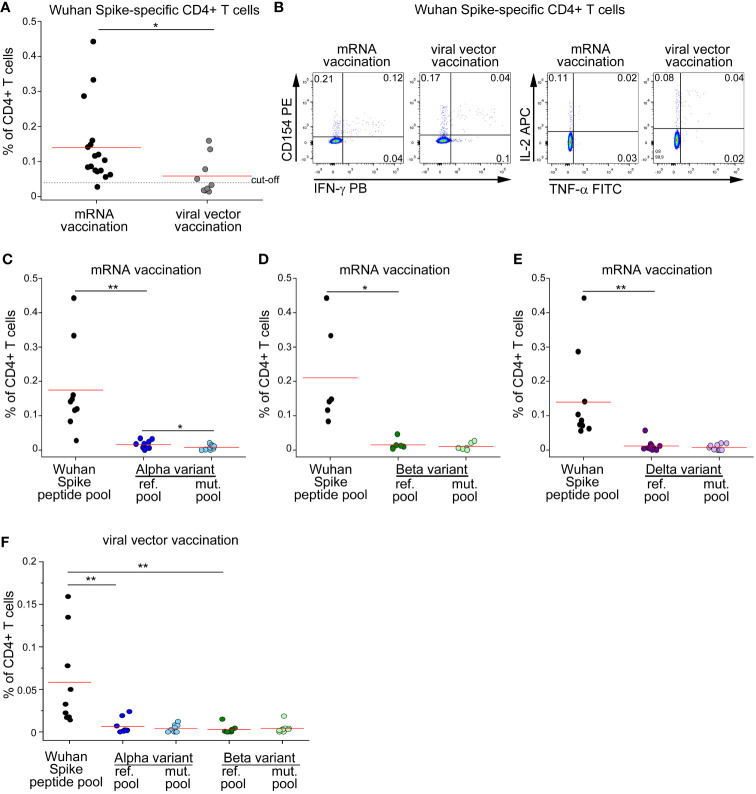
CD4+ T cell response to Spike in vaccinated subjects is minimally affected by mutations occurring in Alpha, Beta and Delta variants. **(A)** Frequency of CD4+ T cells specific for Wuhan Spike protein in mRNA- (17) or viral vector- (9) vaccinated subjects. Representative FACS plot are depicted in **(B)**. Frequency of CD4+ T cells specific for Wuhan Spike, Alpha variant reference or mutated pool **(C)**, Beta variant reference or mutated pool **(D)**, Delta variant reference or mutated pool **(E)** in 9, 6 and 10 mRNA-vaccinated subjects, respectively. **(F)** Frequency of CD4+ T cells specific for Wuhan Spike, Alpha variant reference or mutated pool, Beta variant reference or mutated pool in 9 subjects with viral vector vaccination. Data in **(A, C–F)** are subtracted of background, unstimulated condition. Red lines in **(A, C–F)** represent mean values. Dashed line in **(A)** represents cut-off value. *p < 0.05; **p < 0.01, calculated with Mann-Whitney test **(A)** or Wilcoxon-Signed Rank test **(C–F)**.

Since currently approved vaccines elicit cellular and humoral immunity against ancestral Spike protein, we decided to test whether vaccine-induced CD4+ T cell response is affected by mutations occurring in SARS-CoV-2 variants. We enrolled subjects without prior SARS-CoV-2 infection (defined by absence of compatible symptoms and absence of serum anti-Nucleoprotein IgG), immunized with double dose of either mRNA or viral-vector vaccines. As shown in **Figures 2A, B**, mRNA vaccination elicited significantly higher levels of total Spike-specific CD4+ T cells. As already observed in the context of natural infection, also the frequencies of mRNA- or viral vector-induced CD4+ T cells specific for Alpha or Beta reference pools were significantly lower than those specific for the entire ancestral Spike protein (**Figures 2C, D, F**). When assessing the impact of Alpha and Beta variants on recognition by specific CD4+ T cells, we found a mild impact of Alpha variant on mRNA-induced CD4+ T cell response with no differences in the other contexts (**Figures 2C, D, F**). We also had the possibility to test the impact of Delta variant on vaccine-induced CD4+ T cell response in a cohort of mRNA-vaccinated subjects. Similarly to what observed for Alpha and Beta variants, stimulation with the entire ancestral Spike protein elicited a significantly higher frequency of reactive CD4+ T cells than the Delta reference pool (**Figure 2E**). However, no differences were detected when comparing the frequencies of CD4+ T cells specific for Delta reference or mutated pools (**Figure 2E**). Collectively, these data suggest that vaccine-induced CD4+ T cell response is minimally affected by mutations occurring in Alpha, Beta and Delta variants.

**Figure 3:**
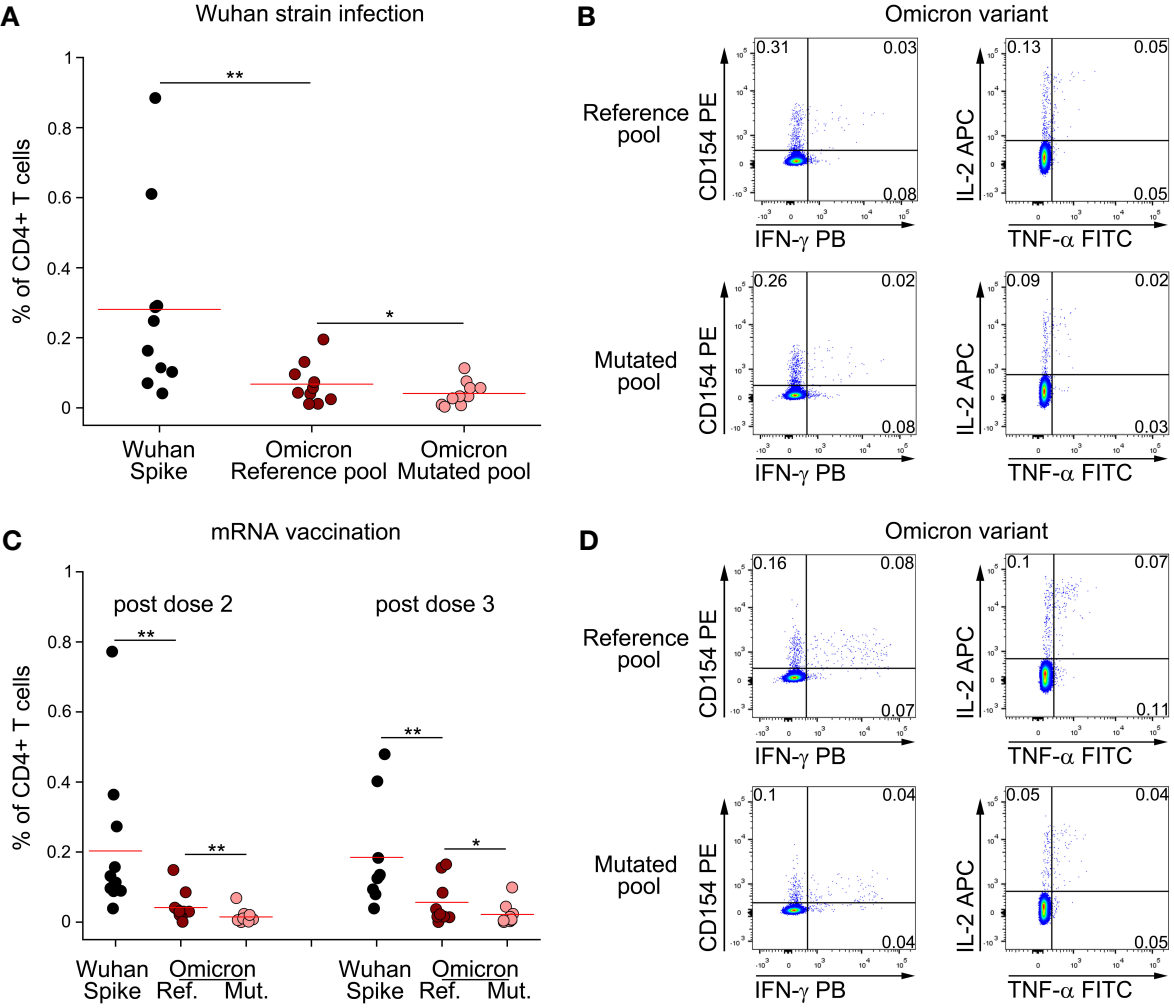
Omicron variant does not significantly impair CD4+ T cell memory response to Spike following natural infection and mRNA vaccination. **(A)** Frequency of CD4+ T cells specific for Wuhan Spike, Omicron variant reference or mutated pool in 10 subjects with previous Wuhan strain SARS-CoV-2 infection. Representative FACS plot are depicted in **(B)**. **(C)** Frequency of CD4+ T cells specific for Wuhan Spike, Omicron variant reference or mutated pool in mRNA-vaccinated subjects enrolled after dose 2 (n=11) or dose 3 (n=9) administration. Representative FACS plot are depicted in **(D)**. Data in **(A, C)** are subtracted of background, unstimulated condition. Red lines in **(A, C)** represent mean value. *p < 0.05; **p < 0.01, calculated Wilcoxon-Signed Rank test.

Following the rapid diffusion of the Omicron variant worldwide, we extended our study to investigate if CD4+ T cell memory response to Spike is affected by mutations present in this viral strain. We stimulated PBMC from subjects who had recovered from ancestral SARS-CoV-2 infection with a peptide pool covering the entire Spike protein, or with reference or mutated peptide pools specific for Omicron variant (**Figures 3A, B**). As previously shown for other variants, CD4+ T cell response against total Spike was significantly higher than Omicron reference pool (**Figure 3A**). Indeed, Omicron reference pool accounted for, on average, 24% of the total response against ancestral Spike. We also observed that mutations present in Omicron affect CD4+ T cell immunity, as we observed a significantly reduced response to the mutated pool when compared to the reference pool (**Figure 3A**). In particular, we found that the mutated pool elicited a 40% lower response when compared to the reference pool. Despite this loss, combining all these information our data suggest that roughly 90% of CD4+ T cell response against Omicron should be preserved with respect to ancestral Spike. We performed the same analysis also on mRNA-vaccinated subjects (**Figures 3C, D**). In this case, we had the possibility to test these subjects one month after dose 2 and dose 3 injections. CD4+ T cell response against total Spike was significantly higher than Omicron reference pool, both after dose 2 and 3 (**Figure 3C**). Omicron reference pool represented 20% and 30% of total CD4+ T cell response against Spike after dose 2 and 3, respectively. When we assessed the impact of Omicron-specific mutations, we observed that the mutated pool elicited a significantly lower response than the reference pool. In particular, we observed a 65% and 61% lower response after dose 2 and 3, respectively. Combining these data, we expect that following dose 2 and 3 of mRNA vaccination, 87% and 82% of total CD4+ T cell response to Spike should be retained against Omicron, respectively.

## Discussion

Our results are in harmony with published data showing that mutations occurring in common SARS-CoV-2 variants have a negligible impact on T cell reactivity (15–17). Notably, these studies were performed stimulating PBMNC with full-length ancestral- or mutated-Spike. Here, we provided the complementary observation that ancestral- or Alpha variant-infection elicit comparable CD4+ T cell responses to ancestral Spike. Notably, this information was obtained from a cohort of COVID-19 patients with a wide range in terms of symptomatology, similar between ancestral- and Alpha variant-infection. This is of importance, as previous studies have shown that disease severity directly correlates to anti-SARS-CoV-2 immunity (13, 14, 18). Our observation that Alpha, Beta and Delta reference pools elicit a CD4+ T cell response significantly lower than total Spike protein provides a biological explanation for the conserved CD4+ T cell response, suggesting that the majority of CD4+ T cells target regions non-mutated in Alpha, Beta and Delta variants. This finding is supported also by a recent publication showing that the overall contribution of T cells targeting mutated regions to the total Spike-specific T cell response is on average 14% for the Alpha variant and 10% for the Beta variant (19). Moreover, despite a mild difference for Alpha variant, we observed substantially comparable responses to reference and mutated pools, suggesting that CD4+ T cells induced by ancestral infection or vaccination cross-react to Alpha, Beta and Delta variants. However, additional studies are needed to fully elucidate this concept. Notably, another research group reported a conserved response for Alpha variant (20). The recent emergence of Omicron variant is worrisome, as this variant exhibits increased transmissibility than Delta and a significant number of mutations in Spike, mainly in the receptor-binding domain. This variant has rapidly become the leading SARS-CoV-2 strain worldwide, with a rapid increase in COVID-19 cases also in highly vaccinated countries (1). Indeed, accumulating data are now showing that the neutralizing capability of convalescent or vaccinees’ sera is significantly reduced against Omicron (8). Regarding T cell immunity, we found that Omicron can significantly blunt the CD4+ T cell response to the regions of Spike targeted by mutations. However, when considering the overall response to Spike, we found that the majority of the response is conserved. Our observations are in agreement with works from other groups recently released as pre-print publications, which suggest minimal escape of T cell immunity by Omicron (21–26). Although it may not be sufficient alone to guarantee protection from infection, a conserved CD4+ T cell immunity against common SARS-CoV-2 VOCs may be fundamental to reduce disease severity, as demonstrated by the importance of a rapid T cell response in preventing severe COVID-19 (27). Notably, our data show that CD4+ T cell response is conserved to variants of concern both in recovered-COVID-19 subjects as well as in vaccinated individuals. Although our cohort included only 25 individuals, we observed that mRNA vaccines elicited significantly higher CD4+ T cell response to Spike than viral vector vaccines. This is in agreement with a previous study which showed that the frequency of CD69+IFN-γ+ CD4+ T cells reactive to Spike is significantly higher following two mRNA vaccine injections than after two viral vector administrations (28). However, despite a significantly different total response to Spike, we found that in both cases the majority of CD4+ T cell response to Spike is preserved also against variants of concern.

We and others have previously demonstrated that following the first mRNA vaccine injection, Spike-specific CD4+ T cells rapidly increase in the circulation of previously infected subjects, with no additional effect following the second dose administration (29, 30). The demonstration that Spike-specific CD4+ T cells from subjects with previous Alpha variant infection recognize also the ancestral protein, suggests that vaccination may rapidly reactivate immunological memory to Spike protein also in these individuals. This is of importance, as in many countries only one vaccine dose is currently administered to individuals with previous COVID-19, regardless of which SARS-CoV-2 variant was responsible of the infection.

In conclusion, our data show that CD4+ T cell memory response to Spike is relatively conserved in the context of Alpha, Beta and Delta SARS-CoV-2 variants. Omicron variant is currently responsible for the vast majority of infections worldwide, due to significant growth advantage and potential immune escape compared to Delta variant (31). Thus, additional information on memory T cell responses to this variant are urgently needed. Our findings obtained following natural infection and in the context of mRNA vaccination suggest that the global CD4+ T cell response is retained also against Omicron. For this reason, vaccination strategies should be implemented to rapidly achieve high immunization levels worldwide, thus conferring protection against moderate-severe COVID-19 caused by SARS-CoV-2 VOCs.

## Materials and Methods

### Subjects

PBMC from 31 moderate COVID-19 patients (18 infected by the original SARS-CoV-2 Wuhan strain and 13 by the Alpha variant) were collected one month following hospital discharge. Alpha-infected subjects were enrolled between April and May 2021. Alpha infection was defined based on presence of N501Y e DEL69/70 mutation, assessed by Allplex SARS-CoV-2 Variants I Assay (Seegene). Main demographic and clinical characteristics are summarized in **Supplementary Table S1**. PBMC from 17 mRNA- and 9 viral-vector-vaccinated subjects were collected one month following the second vaccine dose administration. M:F ratio was 0.54 in the first cohort and 0.5 in the second cohort. Median age (IQR) was 45 (33-58) years for mRNA-vaccinated individuals, and 47 (30-53) years for viral-vector vaccinated subjects. Main demographic and clinical characteristics are summarized in **Supplementary Table S2**. PBMNC from 10 SARS-CoV-2 Wuhan-strain infected subjects were obtained one month after hospital discharge and tested for CD4+ T cell reactivity against Omicron variant. Main demographic and clinical characteristics are summarized in **Supplementary Table S3**. PBMC from 11 mRNA-vaccinated subjects were collected one month after the second and one month after the third dose administration and tested for CD4+ T cell reactivity against Omicron variant. Main demographic and clinical characteristics are summarized in **Supplementary Table S4**.

All the enrolled individuals were of Caucasian origin. PBMC were obtained following density gradient centrifugation of blood samples using Lymphoprep (Axis Shield Poc As™) and were frozen in FCS plus 10% DMSO and stored in liquid nitrogen.

### Evaluation of SARS-CoV-2-Spike-Reactive T Cells

Identification of Spike specific CD4+ T cells was performed as previously described (9, 10). Briefly, 1.5 million thawed PBMNCs were cultured in complete RPMI plus 5% human AB serum in 96 well flat bottom plates in presence of medium alone (background, negative control) or of a pool of Spike SARS-CoV-2 peptide pools (Prot_S1, Prot_S+ and Prot_S to achieve a complete sequence coverage of the Spike protein) at 0.6 µM/peptide, (Miltenyi Biotech). Alternatively, cells were stimulated with peptide pools selectively spanning mutated regions in Alpha, Beta, Delta or Omicron variants and harboring the specific mutations (Alpha, Beta, Delta or Omicron mutated pool, Miltenyi Biotech). As a control, the same peptide pools but with the original (Wuhan strain) sequence were used (reference pool, Miltenyi Biotech). After 2 hours of incubation at 37°C, 5% CO2, Brefeldin A (5 µg/mL) was added, followed by additional 4 hours incubation. Finally, cells were fixed and stained using fluorochrome-conjugated antibodies listed in **Supplementary Table S5**. Samples were acquired on a BD LSR II flow cytometer (BD Biosciences). Cut-off values for CD4+ T cell response to total spike were calculated as mean+2SD from a population of 15 SARS-CoV-2 unexposed, unvaccinated subjects. Gating strategy for the identification of antigen-specific CD4+ T cells is reported in **Supplementary Figure 1**.

### Statistics

Unpaired Mann-Whitney test was used to compare ancestral- versus Alpha variant-SARS-CoV-2 previously infected subjects, as well as mRNA versus viral vector-vaccines. Wilcoxon-Signed Rank test was used to compare the different response to total Spike, reference and mutated pools within each study group. In all cases, p values ≤0.05 were considered significant.

## Data Availability Statement

The original contributions presented in the study are included in the article/**Supplementary Material**. Further inquiries can be directed to the corresponding authors.

## Ethics Statement

The studies involving human participants were reviewed and approved by Careggi University Hospital Ethical Committee. The patients/participants provided their written informed consent to participate in this study.

## Author Contributions

AM, AV, MCa, GL, LS, MCo, AA, NG, NA, FM, LM, AC, and PF performed the experiments. MS and LZ enrolled patients. FL, GMR AB, LC, LM, and FA supervised research. AM and FA wrote the manuscript. All authors contributed to manuscript revision, read, and approved the submitted version.

## Funding

This study was supported by funds to the Department of Experimental and Clinical Medicine, University of Florence, derived from Ministero dell’Istruzione, dell’Università e della Ricerca (Italy) (Project Excellence Departments 2018-2022); by University of Florence, project RICTD2122; by Tuscany Region, project TagSARS-CoV-2; by Ministero della Salute, project COVID-2020-12371849.

## Conflict of Interest

The authors declare that the research was conducted in the absence of any commercial or financial relationships that could be construed as a potential conflict of interest.

## Publisher’s Note

All claims expressed in this article are solely those of the authors and do not necessarily represent those of their affiliated organizations, or those of the publisher, the editors and the reviewers. Any product that may be evaluated in this article, or claim that may be made by its manufacturer, is not guaranteed or endorsed by the publisher.
