# Supplementary material for: SARS-CoV-2 Spike-Specific CD4+ T Cell Response Is Conserved Against Variants of Concern, Including Omicron
Source: Front Immunol. 2022 Jan 26;13:801431. doi: 10.3389/fimmu.2022.801431 (PMC8826050; doi:10.3389/fimmu.2022.801431)

**Supplementary Table S1. Demographic and clinical features of COVID-19 convalescent subjects**

| **Case** | **Type of infection** | **Gender** | **Age** | **COVID-19** | **Comorbidities** |
| --- | --- | --- | --- | --- | --- |
| W1 | Wuhan strain | M | 48 | mild | H |
| W2 | Wuhan strain | M | 51 | mild | PM, aortic valve prosthesis |
| W3 | Wuhan strain | M | 77 | mild | H, T2D, chronic AF |
| W4 | Wuhan strain | F | 88 | mild | H, CAD, COPD, T2D, CKD, obesity, Sjögren’s syndrome |
| W5 | Wuhan variant | F | 55 | mild | H |
| W6 | Wuhan strain | M | 56 | moderate | H |
| W7 | Wuhan strain | M | 56 | moderate | H, T2D, DYS, obesity |
| W8 | Wuhan strain | F | 57 | moderate | T2D |
| W9 | Wuhan strain | F | 87 | moderate | H, stroke, ulcerative colitis |
| W10 | Wuhan strain | M | 49 | severe | H |
| W11 | Wuhan strain | M | 53 | severe | T2D, DYS |
| W12 | Wuhan strain | F | 56 | severe | None |
| W13 | Wuhan strain | M | 58 | critical | None |
| W14 | Wuhan strain | M | 62 | critical | H, T2D, DYS |
| W15 | Wuhan strain | F | 73 | critical | H, COPD, giant cell arteritis, eosinophilic pneumonia |
| W16 | Wuhan strain | F | 75 | critical | H, chronic AF, stroke |
| W17 | Wuhan strain | M | 83 | critical | CAD |
| W18 | Wuhan strain | M | 85 | critical | H, T2D, CKD, CAD, obesity |
| A1 | Alpha variant | M | 42 | moderate | None |
| A2 | Alpha variant | M | 50 | moderate | H, T2D, obesity, PM |
| A3 | Alpha variant | M | 51 | moderate | None |
| A4 | Alpha variant | F | 61 | moderate | None |
| A5 | Alpha variant | M | 61 | moderate | H |
| A6 | Alpha variant | F | 64 | moderate | Hemochromatosis |
| A7 | Alpha variant | M | 44 | severe | H, T2D |
| A8 | Alpha variant | F | 56 | severe | H, asthma, connective tissue disease |
| A9 | Alpha variant | M | 59 | severe | Asthma, psoriasis |
| A10 | Alpha variant | F | 61 | severe | H, chronic HCV infection |
| A11 | Alpha variant | M | 65 | severe | H, COPD, DYS |
| A12 | Alpha variant | F | 68 | severe | H, obesity |
| A13 | Alpha variant | M | 76 | critical | H, T2D, chronic AF, pulmonary embolism |

AF: atrial fibrillation; CAD: coronary artery disease; CKD: chronic kidney disease; COPD: chronic obstructive pulmonary disease; DYS: dyslipidemia; H: hypertension; PM: pacemaker carrier; T2D: type 2 diabetes.

**Supplementary Table S2. Main demographic characteristics of COVID-19 vaccinated subjects**

| **Case** | **Type of vaccine** | **Gender** | **Age** |
| --- | --- | --- | --- |
| R1 | BNT162b2 | M | 20 |
| R2 | BNT162b2 | M | 23 |
| R3 | BNT162b2 | M | 27 |
| R4 | BNT162b2 | F | 30 |
| R5 | BNT162b2 | M | 33 |
| R6 | BNT162b2 | F | 36 |
| R7 | BNT162b2 | F | 38 |
| R8 | BNT162b2 | F | 42 |
| R9 | BNT162b2 | F | 45 |
| R10 | BNT162b2 | F | 47 |
| R11 | BNT162b2 | F | 53 |
| R12 | BNT162b2 | F | 62 |
| R13 | BNT162b2 | M | 82 |
| R14 | mRNA-1273 | F | 46 |
| R15 | mRNA-1273 | F | 58 |
| R16 | mRNA-1273 | M | 64 |
| R17 | mRNA-1273 | F | 72 |
| V1 | ChAdOx1 | F | 26 |
| V2 | ChAdOx1 | F | 26 |
| V3 | ChAdOx1 | M | 30 |
| V4 | ChAdOx1 | F | 30 |
| V5 | ChAdOx1 | M | 47 |
| V6 | ChAdOx1 | F | 53 |
| V7 | ChAdOx1 | F | 53 |
| V8 | ChAdOx1 | M | 60 |
| V9 | ChAdOx1 | F | 66 |

**Supplementary Table S3. Demographic and clinical features of COVID-19 convalescent subjects tested against Omicron variant.**

| **Case** | **Type of infection** | **Gender** | **Age** | **COVID-19** | **Comorbidities** |
| --- | --- | --- | --- | --- | --- |
| OW1 | Wuhan strain | M | 35 | asymptomatic | None |
| OW2 | Wuhan strain | F | 46 | asymptomatic | None |
| OW3 | Wuhan strain | F | 54 | moderate | major depressive disorder |
| OW4 | Wuhan strain | M | 70 | moderate | H, obesity, chronic AF, DYS, pulmonary emphysema |
| OW5 | Wuhan strain | F | 82 | moderate | osteoporosis |
| OW6 | Wuhan strain | F | 82 | moderate | H, T2D, obesity, CKD |
| OW7 | Wuhan strain | M | 64 | severe | H |
| OW8 | Wuhan strain | F | 73 | critical | T2D, DYS |
| OW9 | Wuhan strain | F | 79 | critical | H, T2D, COPD, abdominal aortic aneurism |
| OW10 | Wuhan strain | F | 86 | critical | osteoporosis |

AF: atrial fibrillation; CKD: chronic kidney disease; COPD: chronic obstructive pulmonary disease; DYS: dyslipidemia; H: hypertension; T2D: type 2 diabetes.

**Supplementary Table S4. Main demographic characteristics of COVID-19 vaccinated subjects tested against Omicron variant.**

| **Case** | **Type of vaccine (two doses + booster dose)** | **Gender** | **Age** |
| --- | --- | --- | --- |
| OV1 | BNT162b2 + BNT162b2 | F | 27 |
| OV2 | BNT162b2 + BNT162b2 | F | 56 |
| OV3 | BNT162b2 + BNT162b2 | M | 63 |
| OV4 | BNT162b2 + BNT162b2 | M | 65 |
| OV5 | BNT162b2 + BNT162b2 | F | 23 |
| OV6 | BNT162b2 + BNT162b2 | M | 59 |
| OV7 | BNT162b2 + BNT162b2 | M | 31 |
| OV8 | BNT162b2 + BNT162b2 | F | 59 |
| OV9 | BNT162b2 + BNT162b2 | F | 27 |
| OV10 | BNT162b2 + BNT162b2 | F | 57 |
| OV11 | BNT162b2 + BNT162b2 | F | 55 |

**Supplementary Table S5. List of all fluorochrome mAbs used for flow cytometric analysis of antigen specific T cells.**

| **Antigen** | **Flurochrome** | **Clone** | **Company** |
| --- | --- | --- | --- |
| TNF-α | FITC | 6401.1111 | BDBioscience |
| CD154 | PE | TRAP1 | BDBioscience |
| CD3 | PerCP | SK7 | BDBioscience |
| CD4 | PE-Cy7 | SK3 | Invitrogen |
| CD8 | Super Bright 600 | SK1 | eBioscience™ |
| IL-2 | APC | MQ1-17H12 | BDBioscience |
| IFN-γ | Pacific Blue | B27 | BioLegend |
| L/D | Fixable Viability Stain 780 |  | BDBioscience |

**Supplementary Figure S1. Gating strategy for the identification of Spike-specific CD4+ T cells**

Lymphocytes were identified based on physical parameters (FSC-SSC). Then, we removed doublets by using FSC-A vs FSC-H. Dead cells were then removed by using viability stain 780. Among live cells, we identified lymphocytes based on CD3 expression and then we selected T helper cells based on CD4. Early activated cells were identified by CD154 expression and then further characterized by IFN-γ and TNF-α production. Finally, we monitored IL-2 production by each of the four cell subsets defined by the combinatorial expression of IFN-γ and TNF-α.


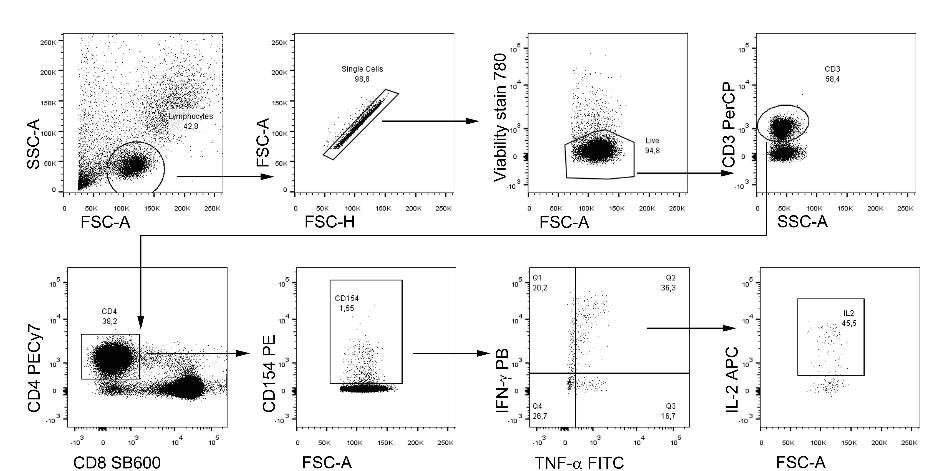

Supplement: Supplementary file 1 [file DataSheet_1.docx]
